# Accelerated hypofractionated three-dimensional conformal radiation therapy (3 Gy/fraction) combined with concurrent chemotherapy for patients with unresectable stage III non-small cell lung cancer: preliminary results of an early terminated phase II trial

**DOI:** 10.1186/s12885-016-2314-1

**Published:** 2016-04-23

**Authors:** Xiao-Cang Ren, Quan-Yu Wang, Rui Zhang, Xue-Ji Chen, Na Wang, Yue-E Liu, Jie Zong, Zhi-Jun Guo, Dong-Ying Wang, Qiang Lin

**Affiliations:** Department of Oncology, North China Petroleum Bureau General Hospital of Hebei Medical University, 8 Huizhan Avenue, Renqiu City, Hebei Province 062552 P.R. China; Department of Radiology, North China Petroleum Bureau General Hospital of Hebei Medical University, 8 Huizhan Avenue, Renqiu, Hebei Province 062552 P.R. China; Department of Cardiovascular Medicine, North China Petroleum Bureau General Hospital of Hebei Medical University, 8 Huizhan Avenue, Renqiu, Hebei Province 062552 P.R. China

**Keywords:** Non-small cell lung cancer, Accelerated hypofractionated radiotherapy, Three-dimensional conformal radiation therapy, Concurrent radiochemotherapy, Maximum tolerated dose

## Abstract

**Background:**

Increasing the biological effective dose (BED) of radiotherapy for non-small cell lung cancer (NSCLC) can increase local control rates and improve overall survival. Compared with conventional fractionated radiotherapy, accelerated hypofractionated radiotherapy can yield higher BED, shorten the total treatment time, and theoretically obtain better efficacy. However, currently, there is no optimal hypofractionated radiotherapy regimen. Based on phase I trial results, we performed this phase II trial to further evaluate the safety and preliminary efficacy of accelerated hypofractionated three-dimensional conformal radiation therapy(3-DCRT) combined with concurrent chemotherapy for patients with unresectable stage III NSCLC.

**Methods:**

Patients with previously untreated unresectable stage III NSCLC received 3-DCRT with a total dose of 69 Gy, delivered at 3 Gy per fraction, once daily, five fractions per week, completed within 4.6 weeks. At the same time, platinum doublet chemotherapy was applied.

**Results:**

After 12 patients were enrolled in the group, the trial was terminated early. There were five cases of grade III radiation esophagitis, of which four cases completed the radiation doses of 51 Gy, 51 Gy, 54 Gy, and 66 Gy, and one case had 16 days of radiation interruption. The incidence of grade III acute esophagitis in patients receiving an irradiation dose per fraction ≥2.7 Gy on the esophagus was 83.3 % (5/6). The incidence of symptomatic grade III radiation pneumonitis among the seven patients who completed 69 Gy according to the plan was 28.6 % (2/7). The median local control (LC) and overall survival (OS) were not achieved; the 1-year LC rate was 59.3 %, and the 1-year OS rate was 78.6 %.

**Conclusion:**

For unresectable stage III NSCLC, the accelerated hypofractionated radiotherapy with a total dose of 69 Gy (3 Gy/f) combined with concurrent chemotherapy might result in severe radiation esophagitis and pneumonitis to severely affect the completion of the radiotherapy. Therefore, we considered that this regimen was infeasible. During the hypofractionated radiotherapy with concurrent chemotherapy, the irradiation dose per fraction to esophagus should be lower than 2.7 Gy. Further studies should be performed using esophageal tolerance as a metric in dose escalation protocols.

**Trial registration:**

NCT02720614, the date of registration: March 23, 2016.

## Background

According to the 2008 global cancer statistics, the morbidity and mortality of lung cancer ranks first worldwide [1]. China also faces a similar situation. According to the 2010 data released by the National Cancer Control Office of the National Cancer Center, the new cases of lung cancer every year totaled approximately 600,000, and the cases of death number approximately 490,000 [2]. For unresectable locally advanced non-small cell lung cancer (NSCLC), concurrent radiochemotherapy is the standard treatment [3, 4]. The classical concurrent radiochemotherapy program uses conventional fractionated radiation with a total dose of 60–66 Gy; however, the local recurrence rate is still as high as 30 % [4]. Studies have suggested that increasing the tumor radiation dose could increase the local control, thus improving survival [5, 6]. However, the RTOG06-17 study using the conventional fractionated regimen showed that, compared with the 60-Gy group, the survival was not increased in the 74-Gy high-dose group [7]. Although the exact reasons were not clear, the very long treatment time (7.4 weeks) in the high-dose group might be one of the reasons [3].

Studies in head and neck squamous cell carcinoma have shown that tumor cells start to accelerate repopulation after 4 weeks of radiotherapy; at this time, the doubling time of tumor cells shortened from 60 days without interference to 4 days. To eliminate this re-proliferation, an extra 0.6-Gy dose was required each day for compensation [8]. This result also partially explained the possible reasons behind the poor effect of conventional fractionated radiotherapy. For NSCLC radiotherapy, after 4 weeks, when the treatment time was increased by 1 day, a 0.45-Gy radiation dose was lost. Therefore, extension of the total treatment time might be a key reason causing the failure of local control [9]. The continuous, hyperfractionated, accelerated radiotherapy (CHART) program continued giving hyperfractionated radiation for 12 days; although the total dose was only 54 Gy, the absolute value of the 2-year survival increases by 9 % compared with the conventional fractionation of 60 Gy (2 Gy/fraction) (29 % vs 20 %, respectively) [10]. Hypofractionated radiotherapy has a dosimetric advantage; it shortens the treatment time, increases the biological effective dose (BED), and can potentially reduce the effect of accelerated repopulation on local failure [6, 11]. Compared with hyperfractionated radiation, hypofractionated radiotherapy has the advantages of convenience, economy, and easy implementation; thus, it has increasingly more clinical applications [12–29].

It has been confirmed that combined radiochemotherapy is better than radiotherapy alone [30], conventional fractionated radiotherapy with concurrent chemotherapy is better than sequential radiotherapy and chemotherapy, and the overall survival (OS) shows benefits with a 5.7 % 3-year OS and a 4.5 % 5-year OS [4]. Similarly, hypofractionated radiotherapy combined with concurrent chemotherapy can also theoretically further increase the efficacy. Experimental research has shown that hypofractionated radiotherapy with concurrent chemotherapy could increase the efficacy [31]. However, due to the concern about the aggressive toxicity of hypofractionated radiotherapy with concurrent chemotherapy, this type of clinical research is relatively rare. The applied fractionated dose and chemotherapy regimens have larger differences, and the optimal program of hypofractionated radiotherapy with concurrent chemotherapy has not been confirmed. We previously conducted a phase I study of hypofractionated radiotherapy (3 Gy/fraction) with concurrent chemotherapy and considered that NSCLC could tolerate the high 69-Gy dose [32]. Base on this finding, we performed the current phase II study to further evaluate the safety and preliminary efficacy of 69-Gy/23-fraction (3 Gy/fraction) hypofractionated radiotherapy with concurrent radiochemotherapy. Only 12 cases were enrolled in this study. Because of the strong esophagus and lung toxicity, this trial was prematurely terminated. The detailed results are reported below.

## Methods

### Inclusion criteria

Patients with previously untreated unresectable stage IIIA or stage IIIB NSCLC (as defined by the 2009 staging standards of the International Union Against Cancer (UICC)) were recruited, who were confirmed pathologically or cytologically. The age range was between 18 and 75 years, the Karnofsky performance status (KPS) score was ≥70, and the expected survival time was ≥3 months. The laboratory examination results showed a neutrophil count ≥2.0 × 10^9^, a hemoglobin level ≥100 g/L, a platelet count ≥100 × 10^9^, and the values of serum creatinine, alanine aminotransferase, aspartate aminotransferase, and total bilirubin were lower than the upper limit of the normal values. The patients did not show abnormal electrocardiogram (ECG) results. Additionally, they did not have other combined diseases that required hospitalization.

### Exclusion criteria

Patients who were pregnant, breastfeeding, had another malignant tumor history (with the exception of patients with cervical carcinoma in situ and non-malignant melanoma skin cancer that had been clinically cured for at least 5 years), could not receive concurrent chemotherapy due to medical reasons, and had superior vena cava syndrome and severe lung diseases that affected lung function were excluded.

This clinical trial was approved by the Ethics Committee of the North China Petroleum Bureau General Hospital of Hebei Medical University. This trial was performed in accordance with the principles of human clinical trials and the Helsinki Declaration (1975 edition and 2000 revised edition). All of the patients signed informed consent before enrollment.

### Patient assessment

Patient assessment was performed within 2 weeks before the start of treatment. The items included a complete medical history, comprehensive physical examination, thoracic, abdominal and head enhanced computed tomography (CT) or head magnetic resonance imaging (MRI), ECG, bronchoscopy, whole-body bone scanning using emission CT (ECT) as suggested by clinical, routine blood tests, and full blood biochemical items.

Patients received a physical examination and routine blood tests every week (if necessary, the frequency could be increased). The full blood biochemical tests and ECG were re-examined before each chemotherapy treatment.

### Research design

This phase II clinical trial was an open-label, single-arm, and safety study. The primary endpoint was the toxicity of the accelerated hypofractionated three-dimensional conformal radiation therapy (69 Gy, 3 Gy/fraction) with concurrent chemotherapy program. The secondary research endpoint included progression-free survival (PFS), median survival time (MST), OS, and local control (LC). A sample size of 30 evaluable patients was determined arbitrarily, if the toxicity induced by the regimen could be tolerated [33].

### Radiotherapy

The three-dimensional conformal technology with accelerated hypofractionated radiotherapy was completed within 4.6 weeks, with a total dose of 69 Gy, delivered at 3 Gy per fraction, once daily, five fractions per week. The chemoradiotherapy treatment scheme is depicted in Table 1.

**Table 1:** Concurrent chemoradiotherapy schema

Concurrent chemoradiotherapy schema						
RT regimen: Weeks 1–5: 3 Gy/f, 1 f/d,5 f/w;						
Week	1	2	3	4	5	
RT	**┃┃┃┃┃**	**┃┃┃┃┃**	**┃┃┃┃┃**	**┃┃┃┃┃**	**┃┃┃**	
Chemotherapy for Patient1-6: NVB (25 mg/m2) d1, d8; CBP,AUC = 5 mg/m1.min on d8, repeated every 28 days						
NVB	**♦**	**♦**			**♦**	**♦**
CBP		**●**				**●**
Chemotherapy for Patient7-12: Paclitaxel(T): 30 mg/m2,Cisplatin(P) 20 mg/m2,weekly,w1-w5.						
TP	**▲**	**▲**	**▲**	**▲**	**▲**	

The specific radiotherapy program has been described in detail in the phase I trial [32]. The limitation conditions of irradiation on important organs were as follows: V20 ≤ 30 %, spinal cord 0 % > 40 Gy, and ≤12 cm esophagus within PTV [24, 27]. Limitation of irradiation to the esophagus was not mandatory, and could be appropriately broadened for the better PTV irradiation.

### Chemotherapy

Chemotherapy was conducted concurrently with radiotherapy. Chemotherapy regimen 1 was as follows: vinorelbine (NVB) was administered by intravenous infusion at a dose of 25 mg/m^2^ on day 1 (d1) and day 8 (d8), and carboplatin (CBP) was administered at a concentration-time curve (AUC) of 5 mg/ml on d8. This treatment was repeated every 28 days. One cycle of chemotherapy was performed concurrently with the radiotherapy. Chemotherapy regimen 2 was as follows: paclitaxel at 30 mg/m^2^ and cisplatin at 20 mg/m^2^ (TP) were administrated every week for 5 weeks continuously.

After radiotherapy was finished, consolidation of chemotherapy using the original regimen was conducted for a maximum of four cycles.

### Supportive care and dose adjustment

The regimen of supportive care and dose adjustment is described in detail in the phase I trial [32].

### Evaluation of short-term efficacy and toxicity

Four weeks after the completion of radiotherapy, the short-term efficacy was evaluated using the thoracic-abdominal spiral CT based on the Response Evaluation Criteria in Solid Tumors, version 1.1, (RECIST 1.1) standard [34]. The Common Terminology Criteria for Adverse Events (CTCAE), version 3.0 issued by the National Cancer Institute/National Institutes of Health (NCI/NIH) was used as the standard for toxicity evaluation [35]. Evaluation was performed each week during the radiotherapy. Adverse events that occurred more than 90 days after the beginning of radiotherapy were classified as late toxicity.

### Follow-up and statistics

A follow-up was conducted every 2 months for the first 6 months after the completion of radiotherapy, every 3 months between 6 months and 2 years, and every 6 months thereafter. All of the statistical analyses were performed using the SPSS 19.0 biostatistical software package or the R3.2.2 statistical software package. The 95 % confidence interval was calculated using the exact binomial test. Regression analysis was performed using logistic regression. The correlation analysis of esophagitis was performed using Spearman’s testing [22]. The survival data were evaluated using the Kaplan-Meier method. The survival time was measured from the initiation of the concurrent radiochemotherapy until death due to any cause or the last follow-up event. Only the first treatment failure was considered as the reason for failure. PFS was defined as survival without local recurrence or distant metastases.

## Results

### Patient condition

From April 2013 to July 2014, 12 patients with previously untreated NSCLC confirmed by pathology and cytology were enrolled in this study; all 12 cases received toxicity and efficacy evaluations. The clinical information of patients is shown in Table 2. The median age was 65 years, and the median KPS score was 80. There were nine cases of squamous carcinoma, two cases of adenocarcinoma and one case of undifferentiated carcinoma. There were four cases with stage IIIA and eight cases with stage IIIB disease. The details of the clinical staging are shown in Table 3. The median gross tumor volume (GTV) was 55.7 cm^3^ (mean, 65.3 cm^3^; range, 7.9–178.0 cm^3^), and the median planning target volume (PTV) was 261.0 cm^3^ (mean, 263.3 cm^3^; 130.3–415.7 cm^3^).

**Table 2:** Patient characteristics

Characteristic	No. of patients		Percentage of
	*N* = 12		Patients (%)
Gender			
Male	11		91.7
Female	1		8.3
Age			
Median		65	
Range		45-75	
Karnofsky performance status			
Median		80	
Range		70-90	
Histology			
Squamous cell carcinoma	9		75.0
Adenocarcinoma	2		16.7
Undifferentiated carcinoma	1		8.3
Stage			
IIIA	4		33.3
IIIB	8		66.7

**Table 3:** Detailed staging for patients with NSCLC

NO.	Location	Stage	TNM	Nodal staging
1	Right upper	IIIA	T2aN2M0	4R, 4 L, 10
2	Right upper	IIIA	T3N2M0	2R, 4R,
3	Left lower	IIIA	T4N0M0	None
4	Left lower	IIIB	T1bN3M0	4R, 4 L
5	Right upper	IIIB	T4N3M0	1R, 2R, 4R, 4 L, 5, 7, 8, 10
6	Right upper	IIIB	T4N3M0	4R, 4 L, 5, 10
7	Right upper	IIIB	T2aN3M0	4R, 10, supraclavicular
8	Right middle	IIIA	T3N2M0	1, 2R, 3A, 4R, 6
9	Right lower	IIIB	T4N2M0	2R, 4R, 5, 7
10	Right upper	IIIB	T4N3M0	4R, 4 L, 7, 10
11	Right upper	IIIB	T4N2M0	2R, 4R, 7, 10
12	Right upper	IIIB	T4N3M0	1R, 2R, 3P, 4R, 5,7

### Compliance

Seven among the 12 cases completed the 69-Gy radiation according to the treatment protocol. Five cases did not complete the protocol due to severe radiation esophagitis, one case suspended the radiotherapy for 16 days (suspension of radiation for more 14 days was considered a major violation of the treatment plan), and the other four cases completed 51 Gy, 51 Gy, 54 Gy, and 66 Gy of radiation. The 95 % unilateral confidence interval of grade III and above esophagitis was ≥18.26 %. The incidence of severe esophagitis (grade III and above) in lung cancer radiotherapy does not have recognized standards [36], especially for hypofractionated concurrent radiochemotherapy [22]. We referenced the mean value, 15 % (6–24 %), reported in literature, which was used as the standard [36]. After hypothesis testing, the results showed a significant difference (*p* = 0.02392). Therefore, we consider the incidence of severe esophagitis in our phase II trial higher than that in general studies. In addition, severe esophagitis significantly affected the completion of radiotherapy, and this radiotherapy regimen was not easy to implement in clinical practice. Therefore, this trial was terminated early after only 12 patients were enrolled in the study. All of the patients who received NC treatment completed 1 cycle of concurrent chemotherapy. Six patients who received weekly TP treatment completed 5, 4, 3, 3, 3, and 3 times of weekly concurrent chemotherapy.

### Non-hematological toxicity

The detailed non-hematological toxicities are shown in Table 4. Five patients had acute grade III radiation esophagitis; which occurred at 30 Gy/10 fractions (No. 5), 36 Gy/12 fractions (No. 9), 45 Gy/15 fractions (No. 10), 39 Gy/13fractions (No. 11), and 45 Gy/15 fractions (No. 12). They were all given supportive measures such as intravenous infusion, antacid, and protection of esophagus and gastric mucosa. Patient No. 5 had severe esophageal pain at 45 Gy/15 fractions; due to the poor effect of narcotic analgesics, the patient could not tolerate and gave up the radiotherapy for a total of 16 days; the patient resumed the radiotherapy after esophagitis was reduced to grade II and finally completed 69 Gy/23 fractions. The other four patients who had grade II radiation esophagitis continued for 7–10 days; although they did not interrupt radiotherapy, they all did not finish the protocol and finally completed 51 Gy, 54 Gy, 51 Gy, and 66 Gy of radiotherapy, respectively.

**Table 4:** Non-hematologic toxicity

Item	Grade I		Grade II		Grade III		Grade IV		Total	
	Case	%	Case	%	Case	%	Case	%	Case	%
Acute										
Radiation esophagitis	2	16.7	2	16.7	5	41.7	0	0	9	75.0
Radiation pneumonia	2	16.7	1	8.3	2	16.7	0	0	5	41.7
Radiation dermatitis	7	58.3	0	0	0	0	0	0	7	58.3
Nausea	3	25.0	5	41.7	2	16.7	0	0	10	83.3
Vomiting	4	33.3	2	16.7	0	0	0	0	6	50.0
Anorexia	2	16.7	4	33.3	2	16.7	0	0	8	66.7
Fatigue	4	33.3	2	16.7	1	8.3	0	0	7	58.3
ALT/ AST	2	16.7	0	0	0	0	0	0	2	16.7
BIL	3	25.0	1	8.3	0	0	0	0	4	33.3
Late										
Lung	2	16.7	2	16.7	1	8.3	0	0	5	41.7
Esophagus	0	0	0	0	1	8.3	0	0	1	8.3

*ALT* alanine aminotransferase, *AST* aspartate aminotransferase, *BIL* bilirubin

After the radiotherapy was completed, the radiation esophagitis of patient No. 5, 9, and 11 recovered rapidly, and significant late-stage reaction was not observed (the follow-up time was 3, 6, and 5, respectively; at the 3-month follow-up time, patient No.5 had already died). Patient No.10 had grade I esophagitis at the completion of radiotherapy; the patient had complete remission after the disease was persistent for 2 months and could resume normal eating (the follow-up time was 3 months). Patient No.12 already had dysphagia before admission and had semi-liquid food before radiotherapy. Esophageal imaging showed extrinsic compression changes and local stenosis. After radiotherapy, the dysphagia was partially relieved but was further aggravated after 3 months of radiotherapy than before radiotherapy. It was evaluated as a late esophageal toxicity of grade II. The disease was further aggravated after 6 months, and the patient could only take liquid food. Esophageal dilation therapy or gastrostomy was required (refused by the patient); thus, it was evaluated as a late esophageal toxicity of grade III. However, the disease evaluation showed that the local control and distant metastasis were relieved (the follow-time was 9 months).

We attempted to analyze factors associated with severe radiation esophagitis. Because of the limitations of the small sample size, only the two most likely associated factors relevant to clinical practice could be analyzed: metastasis of the seventh lymph node (mStation 7) and PTV volume. The results showed that neither mStation 7 nor PTV volume were significantly correlated with severe esophagitis; the z values were 0.001 and 0.000, respectively, and the *p* values were 1.000 and 1.000, respectively. Nevertheless, we observed that five patients with grade III radiation esophagitis all showed mStation 7, while the seven patients who did not exhibit grade III and above esophagitis did not show mStation 7. The Station 7 is adjacent to the esophagus. Lymph node metastasis in this region is easy to induce with a high dose of radiation in the esophagus, thus causing severe esophagitis. Although the regression analysis results did not show remarkable significance, we still propose that mStation 7 might be a risk factor for severe esophagitis. The final conclusion requires confirmation in future studies using large sample sizes.

Among the seven cases that completed the 69 Gy according to the treatment plan, three cases had symptomatic pneumonitis (grade II + III); however, among the five cases for which the protocol was not completed, there was no pneumonitis. The comparison between these two showed a significant difference (*χ*^2^ = 3.935, *P* = 0.047).

Nausea, fatigue, and loss of appetite were observed in most patients. However, these digestive tract symptoms were mild and were successfully alleviated through the administration of appropriate antiemetics and intravenous rehydration without affecting the implementation of chemoradiotherapy. Liver and kidney toxicities were rare. The detailed non-hematological toxicities are shown in Table 4.

### Radiation dose on the esophagus

The detailed information of the radiation of the esophagus is shown in Tables 5, 6 and 7. The mean dose was from 263 Gy to 4,282 Gy, and the maximum dose was from 2,832 Gy to 7,222 Gy. The dose volume parameters are shown in Table 5. The percentage of the esophageal volume that received a 5 Gy or greater radiation dose (V5) was set as the starting point. The dose increments of 5 Gy was used until V69 (the percentage of the esophageal volume that received a 69 Gy or greater radiation dose); a total of 14 dosimetric-volumetric parameters ranging from V5 to V69 were defined. The irradiation dose per fraction parameters are shown in Table 6. The Spearman’s testing results showed that grade III acute esophagitis had a significant positive correlation with the irradiation dose per fraction to the esophagus, the total of 14 dose-volume parameters (V5-V69), maximum radiation dose, and mean radiation dose (*P* < 0.05).

**Table 5:** Irradiation dose to esophagus:^a^ Dose-volume

NO.	V5	V10	V15	V20	V25	V30	V35	V40	V45	V50	V55	V60	V65	V69
	(%)	(%)	(%)	(%)	(%)	(%)	(%)	(%)	(%)	(%)	(%)	(%)	(%)	(%)
1	9.93	1.31	0.52	0	0	0	0	0	0	0	0	0	0	0
2	39.71	29.79	28.22	25.34	18.29	14.63	12.28	11.76	9.93	7.05	3.14	0	0	0
3	31.35	31.35	31.35	31.35	30.31	30.31	30.31	30.31	30.31	30.31	29	25.87	19.60	0.78
4	41.54	38.67	38.67	38.67	24.82	0	0	0	0	0	0	0	0	0
5	65.32	62.97	60.36	58.00	58.00	58.00	57.74	57.22	56.70	55.39	50.95	49.90	41.81	6.01
6	37.10	30.05	27.96	24.56	24.04	23.52	19.33	9.93	3.66	2.87	0	0	0	0
7	30.31	30.31	29.52	19.07	4.96	2.87	2.61	1.57	0	0	0	0	0	0
8	65.06	56.70	53.82	49.38	45.20	33.97	32.66	25.61	4.96	0.52	0	0	0	0
9	44.94	41.54	41.02	40.76	36.58	30.57	20.08	19.33	19.07	18.29	17.51	16.72	15.42	2.35
10	54.09	48.86	45.99	45.46	43.11	42.85	37.89	37.89	34.44	34.23	32.40	28.74	17.24	0
11	40.12	38.93	38.64	36.84	35.80	33.97	32.40	31.62	29.52	28.48	28.22	26.39	26.13	20.12
12	70.02	66.63	65.84	64.54	64.28	64.28	64.01	63.75	61.62	61.40	58.27	53.82	47.03	18.81
*p*	*0.019*	*0.010*	*0.019*	*0.019*	*0.010*	*0.002*	*0.009*	*0.004*	*0.004*	*0.004*	*0.002*	*0.000*	*0.001*	*0.006*
*r* ^*b*^	*0.661*	*0.710*	*0.661*	*0.661*	*0.710*	*0.786*	*0.711*	*0.760*	*0.764*	*0.764*	*0.787*	*0.862*	*0.810*	*0.737*

^a^The cell values demonstrate the percent of total esophagus volume receiving a dose or greater than a certain dose. For example, “V50 = 29 %” demonstrated that the esophagus volume received 50Gy or more was 29 %

^b^“*r*” refers to the correlation coefficient calculated by Spearman’s testing

**Table 6:** Irradiation dose to esophagus:^a^ Dose per fractionation

NO.	2 Gy	2.2 Gy	2.4 Gy	2.6 Gy	2.7 Gy	2.8 Gy	2.85 Gy	2.9 Gy	2.95 Gy	3 Gy
	cm	cm	cm	cm	cm	cm	cm	cm	cm	cm
1	0	0	0	0	0	0	0	0	0	0
2	3	2	0	0	0	0	0	0	0	0
3	8.5	8.5	8.5	7.5	7	6.5	5.5	5	2.5	2
4	0	0	0	0	0	0	0	0	0	0
5	14	14	13.5	13.5	13	12.5	12	10.5	8	4
6	5.5	4.5	4	1	0	0	0	0	0	0
7	2	0	0	0	0	0	0	0	0	0
8	6	5	1.5	0	0	0	0	0	0	0
9	5.5	5.5	4.5	4.5	4.5	4.5	3.5	3	3	2
10	8.5	8.5	8.5	8	7.5	7	6.5	5	0	0
11	7	7	6	6	6	6	6	6	6	6
12	12.5	12.5	12	11	11	11	11	11	7.5	5.5
*p*	*0.018*	*0.006*	*0.005*	*0.002*	*0.001*	*0.001*	*0.000*	*0.001*	*0.006*	*0.009*
*r* ^*b*^	*0.665*	*0.741*	*0.749*	*0.787*	*0.810*	*0.810*	*0.862*	*0.838*	*0.737*	*0.711*

^a^The cell values demonstrate the whole circumferential length of esophagus receiving a dose greater than a certain dose per fractionation. For example, “2.7Gy = 13 cm” demonstrated that the whole circumferential length of esophagus received 2.7Gy per fraction or more was 13 cm

^b^“*r*” refers to the correlation coefficient calculated by Spearman’s testing

**Table 7:** Irradiation dose to esophagus: Maximum and mean

NO.	Maximum (Gy)	Mean (Gy)
1	2832	263
2	5793	1183
3	7023	2029
4	2887	1063
5	7203	3916
6	5839	1181
7	4510	766
8	5045	2092
9	6992	2025
10	6892	2748
11	7004	2304
12	7222	4282
*p*	*0.010*	*0.004*
*r* ^a^	*0.710*	*0.759*

^a^“*r*” refers to the correlation coefficient calculated by Spearman’s testing

### Hematological toxicity

Neutropenia occurred in 58.3 % (7/12) of patients; the overall disease was milder, and only one case had agranulocytosis (8.3 %). The percentage of the decrease of platelets and hemoglobin was low, and grade II and above disease did not occur. The hematological toxicity of weekly TP treatment was significantly milder than that in NC chemotherapy; the percentages of neutropenia were 16.7 % and 100 %, respectively. The detailed hematological toxicities are shown in Table 8.

**Table 8:** Hematologic toxicity

Item	Grade I		Grade II		Grade III		Grade IV		Total	
	Case	%	Case	%	Case	%	Case	%	Case	%
Neutropenia	1	8.3	3	25.0	1	8.3	1	8.3	6	50.0
Thrombocytopenia	3	25.0	0	0	0	0	0	0	3	25.0
Anemia	2	16.7	0	0	0	0	0	0	2	16.7

### Short-term treatment efficacy

Evaluation of the short-term treatment efficacy was performed on 12 cases. The results showed that the complete response (CR) was 0 % (0/12), the partial response (PR) was 61.5 % (11/12), and the stable disease (SD) was 8.3 % (1/12). There was no progressive disease (PD), and the total response rate (RR) was 91.7 % (11/12).

### Survival

Although this phase II trial was prematurely terminated, we still reported the preliminary survival results. Because the follow-up time was short (5–16 months), the median follow-up was 10 months, there were only two cases of death, and the survival information of OS was not mature. The median PFS, OS and LC, were not reached. The mean PFS, OS, and LC were 12.3, 14.3, and 12.9 months, respectively. The 1-year PFS, OS, and LC were 58.3 %, 78.6 %, and 64.2 %, respectively. There were four cases of treatment failure with two cases of simple local progression, one case of local progression plus distant metastasis (metastasis in both lung), and one case of simple distant metastasis (multiple ipsilateral lung metastasis). Regarding the cause of the two deaths, one case was due to local progression, and one case was due to local progression plus distant metastasis.

## Discussion

The treatment failure of the primary lesions of NSCLC had negative effects on PFS, metastasis-free survival, and OS [37]. Increasing the tumor radiation dose could increase the local control and improve survival [5, 38]. A study using model analysis obtained the same conclusion: the radiotherapy dose and survival had a significant dose-effect relationship, and a high radiotherapy dose could obtain a better 2-year PFS [8]. However, only increasing the radiotherapy dose alone was not sufficient. RTOG0617 used 74 Gy for conventional radiotherapy (concurrent with chemotherapy), and the total treatment time was as long as 7.4 weeks; the results showed that OS was not improved [7]. The other key factor for the radiotherapy efficacy was the total treatment time [10, 11]. Shortening the total treatment time could increase the BED [11]. Application of the hypofractionated radiotherapy not only could obtain higher BED but also could shorten the total treatment time [36]. Therefore, compared with hyperfractionated radiotherapy, it might obtain more benefits [8].

Many studies have reported implementing hypofractionated radiotherapy on unresectable early- and middle-stage NSCLC (IA-IIB); the results showed that there was no severe esophagus and lung toxicities, and the survival results were inspiring [12–15]. Hypofractionated radiotherapy (with or without sequential chemotherapy) on locally advanced NSCLC was also safe and effective. Concerning the toxicity of hypofractionated radiotherapy, the single fractionated dose was rarely above 3 Gy/fraction [16–21]. Radiotherapy with 55 Gy/20 fraction and 2.75 Gy/fraction was extensively applied in the UK. Din et al. [39] retrospectively analyzed 609 cases of hypofractionated radiotherapy, and the results showed that there were 227 cases of IIIA/IIIB, the MST was 20 months, the 2-year OS was 40 %, and toxicity could be tolerated. This regimen could also be implemented safely in the elderly population over the age of 80 years [18].

Conventional fractionated radiotherapy with concurrent chemotherapy was better than simple radiotherapy or sequential radiochemotherapy [3, 4]. Theoretically, it was hypothesized that hypofractionated radiotherapy with concurrent chemotherapy could reasonably further increase efficacy. Therefore, studies exploring hypofractionated radiotherapy with concurrent chemotherapy are emerging [22–29]. These studies obtained inspiring survival results: the MST was 13.4–29.5 months, the 1-year OS was 56–90 %, and the 2-year OS was 30–58.3 % [24–29]. However, except for two studies [24, 27], all other studies had a small sample size and were single-group and phase I/II trials; the number of cases was small with only 10–37 participants. Therefore, these survival results must be confirmed by phase III randomized controlled trials. Although the multivariate analysis in a non-randomized retrospective study showed that radiochemotherapy was the only survival predictive factor [40], but two prospective studies did not confirm that concurrent radiochemotherapy was better than sequential radiochemotherapy [24, 27]. EORTC 08912–22973 was a randomized controlled trial with the most cases; it enrolled 158 patients and used the radiotherapy regimen of 66 Gy/24 fractions and 2.75 Gy/fraction. The concurrent chemotherapy used a low dose of cisplatin at 6 mg/m^2^ every day. The MST, 2-year OS, and 3-year OS in the concurrent radiochemotherapy group and sequential radiochemotherapy group were 16.5 months and 16.2 months, 39 % and 34 %, and 34 % and 22 %, respectively; there were no significant differences [27]. The Sequential or Concurrent Chemotherapy and Radiotherapy (SOCCAR) trial enrolled 130 cases. The radiotherapy regimen was 55 Gy/20 fractions and 2.75 Gy/fraction. The chemotherapy was the vinorelbine + cisplatin regimen. Although both groups obtained good survival results, there was no significant difference. The MST, 1-year OS, and 2-year OS in these two groups were 24.3 months and 18.4 months, 70 % and 83 %, and 50 % and 46 %, respectively [24]. The hypofractionated regimens used in the above studies had very large differences; the fractionated dose ranged from 2.4 Gy/fraction to 3.5 Gy/fraction, the total dose ranged from 52.5 Gy to 66 Gy, and the total treatment time ranged from 26 days to 37 days, and the results were different; therefore, it was difficult to choose the best regimen from these results.

Our previous study performed dose escalation of the 3-Gy/fraction radiotherapy. The maximum-tolerated dose (MTD) recommended in the phase II trial was 69 Gy/23 fractions [32]. However, currently, our phase II trial only enrolled 12 cases. Due to the aggressive esophageal toxicity (mainly esophageal pain) and lung toxicity, the trial was prematurely terminated. The percentage of grade III acute esophagitis in our study was far higher than those in other hypofractionated reports and reached 41.7 % (5/12). Five cases presented with intolerable esophageal pain, and 80 % (4/5) had different degrees of dysphagia, of which one case had very severe dysphagia and could only drink a small amount of water. It was considered that the reasons for the development of such severe esophagitis might be associated with the following factors. First, the total dose was 69 Gy, and the fractionated dose was 3 Gy/fraction in our radiotherapy regimen. The radiation on the esophagus regardless of the total dose or single dose might both be very high [41, 42]. In addition, since the single fractionated dose was large, the rapidity of dose accumulation on the esophagus might cause severe esophagitis [36]. Second, the whole group had 41.7 % lymph node metastasis with more than four stations and 50 % N3 lesions, thus causing extensive involvement of the mediastinum. Mediastinal infiltration and extensive lymph node metastasis are factors for the high incidence of esophagitis [16]. Third, 41.7 % patients had Station 7 metastasis. Station 7 was adjacent to the esophagus; therefore, it was easy to cause the high-dose radiation on esophagus. In our study, the occurrence of esophagitis was early; the earliest case (No. 5) had already developed the complication at the 10^th^ fraction. Although the corresponding measures such as narcotic analgesic drug administration and nutrition support were provided, the grade III esophagitis persisted for 23 days before being reduced to grade II, thus causing 16 days of radiotherapy interruption. This patient had eight stations of mediastinal lymph node metastasis—1R, 2R, 4R, 4 L, 5, 7, 8, and 10. The V60 reached 49.9 %. The whole circumferential length of esophagus receiving more than 2.7 Gy and 3 Gy per fraction reached 13 cm and 4 cm, respectively. Regardless of the total radiation dose, irradiation length, dose-volume percentage, or irradiation dose per fraction to esophagus, they were all very high.

The dosimetric parameters of the occurrence of radiation esophagitis might be associated with the maximum radiation dose, irradiation length, and dose-volume parameters of the esophagus [41–45]. Our study also confirmed these results. However, there were also some conflicting studies considering that these parameters did not have a clear correlation with esophagitis [46, 47]. The dosimetric parameters of radiation esophagitis could not draw a very firm conclusion [36], particularly for hypofractionated radiotherapy with concurrent chemotherapy [22].

When the five cases of grade III esophagitis occurred in our study, the completed radiation dose was only 43 %, 52 %, 57 %, 57 %, and 65 % of the plan. In addition, at the end, they did not complete the radiation of the total dose (with the exception of the patient who had a 16-day interruption). Therefore, the maximum radiation dose and dose-volume parameters of the esophagus in these patients might not be the most relevant factors to predict severe esophagitis [36]. Studies have shown that severe esophagitis was closely associated with the rapid accumulation of the radiation dose of the esophagus, a finding that might be more important than the final completed total irradiation dose [36]. A single large-dose radiation on the esophagus would induce the rapid increase of the radiation dose of the esophagus. Our study also observed this condition: the percentage of severe esophagitis in patients who received one single dose per fraction of ≥2.7-Gy radiation on the esophagus was markedly higher than in those who received a dose lower than 2.7 Gy (80 % vs 0, respectively). Although there was no significant difference, it was considered that the cause might be due to the very small sample size. Therefore, we considered that the severe acute radiation esophagitis might be closely associated with the irradiation dose per fraction to esophagus ≥2.7 Gy.

Other hypofractionated radiotherapy studies also exhibited similar phenomena [26, 27]. Koukourakis et al. applied the 3.5 Gy/fraction concurrent radiochemotherapy, although there was a routine 9-day interval, and also applied the cytoprotective adjuvant amifostine; however, the grade III esophagitis still reached 22.5 % [26]. A study that applied 2.75 Gy/fraction had 17 % grade III/IV esophagitis [27]. In these two high single-dose hypofractionated studies, the percentages of esophagitis were significantly higher than those in hypofractionated studies using a relatively smaller single dose [25, 28, 29]. EORTC 08912 [23] applied a large fractionated dose of 2.75 Gy/fraction; 17 cases received a total radiation dose >60 Gy of the esophagus that was one single irradiation dose of >2.5 Gy/fraction, and the mean length reached 11.4 cm. However, there were only two cases of grade III esophagitis; the radiation doses of the esophagus of these two cases were 65 Gy and 66 Gy, respectively—that is, the single irradiation doses were 2.71 Gy and 2.75 Gy, respectively. These results were consistent with our study results suggesting that the single dose equal to or larger than 2.7 Gy would induce severe esophagitis. Research in the Netherlands reported two cases of grade IV esophageal toxicity of which the radiation dose of the esophagus in one case was 66 Gy/27 fraction and 2.75 Gy/fraction [40]. The above analyses supported the following conclusion. A single dose radiation per fraction ≥2.7 Gy in hypofractionated radiotherapy with concurrent chemotherapy might induce severe radiation esophagitis. In the three studies applying relatively small fractionated doses (2.5 Gy, 2.4 Gy, and 2.4 Gy, respectively), two studies did not have grade III or higher esophagitis [25, 29], and the other one only had 3 % of grade III esophagitis [28]. However, the number of cases in these studies was small, and the number of severe esophagitis cases was even smaller; therefore, this phenomenon was not a confirmed conclusion. In our study, among the five cases of grade III acute esophagitis, only one case was finally transformed into late grade III esophageal injury, indicating that 69 Gy might not cause a severe late esophageal toxicity. However, this result should be treated cautiously because only one case among these five cases completed 69 Gy of radiation (the radiotherapy was interrupted for 16 days due to esophagitis). This case showed rapid disease progression and died after 4 months of completion of the radiotherapy; the observation time was short and was not sufficient to exclude the possibility of the occurrence of grade III and above late esophageal injury. Three of other four cases completed only 74–78 % of the radiation dose; therefore, it could not verify the safety of the 69 Gy of radiation.

In our study, two cases of grade III radiation pneumonitis (16.7 %) transformed into one case of late grade III lung injury and one case of late grade II lung injury after 90 days. It was worth noting that patients who completed 69 Gy of radiation according to the plan had 28.6 % grade III radiation pneumonitis (2/7), and the actual incidence of lung injury might be underestimated (one case died due to disease progression after 4 months of the completion of radiotherapy; thus, the evaluation time for lung injury was short). Currently, there are no established data to guide the prevention and reduction of the development of radiation pneumonia during hypofractionated radiotherapy. Studies have shown that application of hypofractionated radiotherapy using the dose parameters obtained mainly from conventional hypofractionated radiotherapy might induce severe lung toxicity [48, 49]. Therefore, the possibility of developing severe radiation pneumonia during hypofractionated radiotherapy is highly vigilant.

In our study, at the median 10-month follow-up, as high as 41.7 % grade III acute esophagitis and 28.6 % grade III acute pneumonitis (patients who completed the radiotherapy plan) were already observed. The late lung toxicity might be underestimated because of short follow-up time [48]. Because a late lung toxicity was usually fatal [48, 49], we considered that this hypofractionated radiochemotherapy regimen was not safe. In addition, grade III esophagitis in this study presented prominently esophageal pain; thus, 33.3 % patients in this group could not complete the whole 69-Gy radiotherapy, and the compliance of this regimen was poor. Therefore, this phase II trial was terminated early.

In our phase I study, the 69-Gy group enrolled six patients. There were only two cases of grade II esophagitis, and there was no grade III and above esophagitis. The esophageal toxicity was significantly lower than that in the current study [32]. We reviewed the dosimetric parameters of the six patients and found that the maximum total doses of the esophagus of all patients were all ≤55.2 Gy—that is, the maximum single dose per fraction of radiation was ≤2.4 Gy. In the phase II study, the maximum irradiation dose per fraction to esophagus of four patients was ≤2.4 Gy, and there was no grade III esophagitis, findings that were consistent with the result of the hypofractionated concurrent radiochemotherapy using a relatively small single dose [25, 28, 29]. In our phase I study, the 69-Gy group did not have grade III radiation pneumonitis. The latter finding was considered to be associated with the selection bias caused by the small number of cases. A similar phenomenon was also observed in a Canadian report. The maximum single dose per fraction of the dose-escalation was 3.24 Gy/fraction, the total dose was 70.7 Gy, the concurrent full-dose etoposide/cisplatin (EP) chemotherapy regimen was conducted for 2 cycles, and the median 22-month follow-up showed no grade III and above toxicity. Because there were only 10 patients in the group, the authors considered that the results should be cautiously treated with optimism because of the small sample size [25]. Therefore, if the case number in the dose-escalation group was small, the result was not sufficient to exhibit the safety of this dose level, particularly with high-dose hypofractionated radiation [48]. The survival data in our study are still not mature, and the MST was not achieved; however, for the 1-year OS of 78.6 %, these results were comparable to those of other literature reports (OS from 56 % to 90 %) [24–29].

The studies on conventional fractionated radiotherapy in NSCLC focused heavily on lung toxicity However, they did not focus on the protection of the esophagus, and some studies did not even have limitation of irradiation dose to esophagus [28, 29, 50]. Currently, the predictive factors and dose-volume parameters for esophagitis still cannot provide a confirmed conclusion, and the hypofractionated radiotherapy even requires new dosimetric parameters and other factors for evaluation [36]. Although esophagitis, particularly acute esophagitis, is not fatal, it might affect the completion of the radiotherapy plan, interrupt radiotherapy, or decrease the radiation dose, thus reducing the local control rate. This situation is even more prominent in hypofractionated radiotherapy. Some studies have considered that the major obstacle of dose-escalation in hypofractionated radiotherapy was caused by the esophagus [37, 40, 48], a finding that was also confirmed in our phase II study. Therefore, during hypofractionated radiotherapy, particularly concurrent radiochemotherapy, in addition to lung toxicity, acute esophageal toxicity should also be given close attention. The toxicity analysis in hypofractionated concurrent radiochemotherapy showed that the traditional dosimetric parameters did not have a good correlation with esophagitis [22]. Therefore, exploring new predictive factors for esophagitis, particularly for late esophageal injury, has very important clinical significance.

Most radiotherapy studies used the unified prescription dose. However, the application of a fixed dose to all patients might have two consequences. First, some patients might receive a very low radiotherapy dose with insufficient treatment intensity, while other patients might receive a very high radiotherapy dose with strong side effects. The so-called “*in silico*” dose prescription refers to the radiation dose limitation of normal tissues being set up in advance and is used as a standard to allow each patient to receive individualized different maximum radiation doses without increasing toxicity; thus, the treatment intensity is sufficient and safe; this dose is also called the “isotoxic” prescription dose [37, 50–52]. In hyperfractionated studies, the application of this method effectively allows escalation of the radiotherapy dose; in addition, the mature survival results showed that the MST at the IIIB phase reached 17.2 months. This series of studies only conducted limitations of radiation dose on the lung and spinal cord [50–52]. Hoffmann et al. [37] applied the *in silico* method to perform a study on individualized dose prescription for hypofractionation and pre-set the limits of radiation dose on the esophagus, lung, spinal cord, heart, and brachial plexus. The results of the model analysis showed that 79 % of cases had a therapeutic gain in dose escalation. During the pre-set of the radiation dose for normal tissues, the “physical dose” was transformed into the “biological effective dose”; thus, it was more in line with the radiation injury reaction of normal tissues after radiation and might be beneficial for reducing toxicity [16, 25].

Modern advance radiotherapy technologies, including intensity-modulated radiation therapy (IMRT), image-guided radiation therapy (IGRT), 4D-CT, breathing adapted radiotherapy, particle radiotherapy [53], and tomotherapy [54], can more accurately irradiate tumors; at the same time, the radiation dose of surrounding normal tissues can be reduced. Using the *in silico* method combined with advanced radiotherapy technology to perform radiotherapy with an individualized maximum dose based on the isotoxicity of normal tissues can theoretically achieve the dose-escalated hypofractionated concurrent radiochemotherapy more safely.

## Conclusion

In summary, although our hypofractionated concurrent chemoradiotherapy regimen (69 Gy/23 fractions, 3 Gy/fraction) showed preliminary efficacy, it resulted in aggressive esophagus and lung toxicity; particularly, severe esophagitis significantly affected the completion of radiotherapy. Therefore, this regimen was not feasible. Although, it was not the final conclusion, our study suggested that an irradiation dose per fraction to esophagus during hypofractionated concurrent radiochemotherapy should be controlled to be below 2.7 Gy. Currently, we are performing radiation dose escalation in hypofractionated radiochemotherapy of NSCLC using the *in silico* method according to different irradiation doses per fraction to the esophagus.

### Ethics approval and consent to participate

This trial was performed in accordance with the principles of human clinical trials and the Helsinki Declaration (1975 edition and 2000 revised edition). This trial was approved by the Ethics Committee of the North China Petroleum Bureau General Hospital of Hebei Medical University.

All participants signed informed consent before enrollment.

## Abbreviations

3D-CRT: Three-dimensional conformal radiation therapy
CR: Complete response
hypoRT: Hypofractionated radiation therapy
KPS: Karnofsky performance status
NC: Vinorelbine and carboplatin
NSCLC: Non-small cell lung cancer
PD: Progressive disease
PR: Partial response
SD: Stable disease
TP: Paclitaxel and cisplatin
